# Anxiety, concerns and COVID-19: Cross-country perspectives from families and individuals with neurodevelopmental conditions

**DOI:** 10.7189/jogh.13.04081

**Published:** 2023-07-28

**Authors:** Vassilis Sideropoulos, Jo Van Herwegen, Ben Meuleman, Michael Alessandri, Faisal M Alnemary, Jamal Amani Rad, Pamela A Banta Lavenex, Nikita Bolshakov, Sven Bölte, Paulina Buffle, Ru Y Cai, Ruth Campos, Adela Chirita-Emandi, Andreia P Costa, Floriana Costanzo, Vincent Des Portes, Daniel Dukes, Laurence Faivre, Nawelle Famelart, Marisa H Fisher, Liudmilla Gamaiunova, Aikaterini Giannadou, Rashmi Gupta, Antonio Y Hardan, Françoise Houdayer-Robert, Lenka Hrncirova, Roberto Tadeu Iaochite, Katarina Jariabkova, Bonita P Klein-Tasman, Pierre Lavenex, Supriya Malik, Francesca Mari, Pastora Martinez-Castilla, Deny Menghini, Heather J Nuske, Olympia Palikara, Anouk Papon, Robin S Pegg, Hamidreza Pouretemad, Luise Poustka, Ingolf Prosetzky, Alessandra Renieri, Sinead M Rhodes, Deborah M Riby, Massimiliano Rossi, Saeid Sadeghi, Xueyen Su, Claire Tai, Michel Tran, Fionnuala Tynan, Mirko Uljarević, Amy V Van Hecke, Guida Veiga, Alain Verloes, Stefano Vicari, Sonja G Werneck-Rohrer, Eric Zander, Andrea C Samson

**Affiliations:** 1 Department of Psychology and Human Development, IOE, UCL’s Faculty of Education and Society, London, UK; 2 Swiss Center for Affective Sciences, University of Geneva, Geneva, Switzerland; 3 Department of Psychology, University of Miami, Coral Gables, Florida, USA; 4 Autism Center of Excellence, Riyadh, Kingdom of Saudi Arabia; 5 Department of Cognitive Modeling, Institute for Cognitive and Brain Sciences, Shahid Beheshti University, Tehran, Iran; 6 Faculty of Psychology, UniDistance Suisse, Brig, Switzerland; 7 Independent researcher; 8 Center of Neurodevelopmental Disorders at Karolinska Institutet (KIND), Center for Psychiatry Research, Department of Women's and Children's Health, Stockholm, Sweden; 9 Laboratory of Sensori-Motor Affective and Social Development, Faculty of Psychology and Educational Sciences, University of Geneva, Geneva, Switzerland; 10 Aspect Research Centre for Autism Practice, Autism Spectrum Australia, Frenchs Forest, New South Wales, Australia; 11 Department of Basic Psychology, Faculty of Psychology, Universidad Autónoma de Madrid, Madrid, Spain; 12 Department of Microscopic Morphology – Genetics, Center of Genomic Medicine, University of Medicine and Pharmacy “Victor Babes” Timisoara, Timisoara, Romania; 13 Department of Behavioural and Cognitive Sciences, University of Luxembourg, Luxembourg; 14 Child and Adolescents Neuropsychiatry Unit, Bambino Gesù Children's Hospital, Rome, Italy; 15 National Reference Center for Intellectual Disabilities of Rare causes, Lyon University Hospital, France; 16 Institute of Special Education, University of Fribourg, Fribourg, Switzerland; 17 Centre de Génétique et Centre de Référence Anomalies du Développement et Syndromes Malformatifs, FHU TRANSLAD, INSERM UMR1231, Hôpital d’Enfants, CHU Dijon, Dijon Cedex, France; 18 Laboratory CLLE, University of Toulouse, CNRS, Toulouse, France; 19 Department of Counselling, Educational Psychology, & Special Education, Michigan State University, East Lansing, Michigan, USA; 20 Institute for Social Sciences of Religions, University of Lausanne, Lausanne, Switzerland; 21 Cabinet office, London, UK; 22 Cognitive and Behavioural Neuroscience Laboratory, Department of Humanities and Social Sciences, Indian Institute of Technology Bombay, Mumbai, Powai, Maharashtra, India; 23 Department of Psychiatry and Behavioural Sciences, Stanford University, Stanford, California, USA; 24 Genetics Department, Reference Centre for Developmental Disorders Centre East, Bron, France; 25 Department of Special and Inclusive Education, Faculty of Education, Masaryk University, Brno, Czech Republic; 26 Department of Education, Sao Paulo State University (UNESP), Rio Claro, Brazil; 27 Institute for Research in Social Communication, Slovak Academy of Sciences, Bratislava, Slovakia; 28 University of Wisconsin-Milwaukee, Milwaukee, Wisconsin, USA; 29 Laboratory of Brain and Cognitive Development, Institute of Psychology, University of Lausanne, Lausanne, Switzerland; 30 eMbrace, New Delhi, India; 31 Medical Genetics, University of Siena, Siena, Italy; 32 Department of Developmental and Educational Psychology, Universidad Nacional de Educación a Distancia (UNED), Madrid, Spain; 33 Penn Center for Mental Health, University of Pennsylvania Perelman School of Medicine, Philadelphia, Pennsylvania, USA; 34 Department of Education Studies, Faculty of Social Sciences, University of Warwick, Warwick, UK; 35 Williams Syndrome Association, Troy, Michigan, USA; 36 Department of Cognitive Psychology, Institute for Cognitive and Brain Sciences, Shahid Beheshti University, Tehran, Iran; 37 Department of Child and Adolescent Psychiatry and Psychotherapy, University Medical Centre Göttingen, Göttingen, Germany; 38 Faculty of Social Sciences, University of Applied Sciences Zittau / Görlitz, Görlitz, Germany; 39 Centre for Clinical Brain Sciences, University of Edinburgh, Edinburgh, UK; 40 Department of Psychology, Centre for Neurodiversity & Development, Durham University, Durham, UK; 41 Service de génétique HCL, INSERM U1028, CNRS UMR5292, CRNL, GENDEV Team, UCBL1, Bron, France; 42 Department of Early Childhood Education, Faculty of Education, East China Normal University, Shanghai, China; 43 Faculty of Education, Mary Immaculate College, Limerick, Ireland; 44 Department of Psychology, Marquette University, Milwaukee, Wisconsin, USA; 45 Comprehensive Health Research Centre (CHRC), Departamento de Desporto e Saúde, Escola de Saúde e Desenvolvimento Humano, Universidade de Évora, Évora, Portugal; 46 Department of Genetics, APHP.NUP Robert DEBRE University Hospital, Paris, France; 47 Department of Life Sciences and Public Health, Catholic University, Rome, Italy; 48 Department of Child and Adolescent Psychiatry, Medical University of Vienna, Vienna, Austria

## Abstract

**Background:**

The COVID-19 pandemic had a major impact on the mental health and well-being of children with neurodevelopmental conditions (NDCs) and of their families worldwide. However, there is insufficient evidence to understand how different factors (e.g., individual, family, country, children) have impacted on anxiety levels of families and their children with NDCs developed over time.

**Methods:**

We used data from a global survey assessing the experience of 8043 families and their children with NDCs (mean of age (m) *=* 13.18 years, 37% female) and their typically developing siblings (m *=* 12.9 years, 45% female) in combination with data from the European Centre for Disease Prevention and Control, the University of Oxford, and the Central Intelligence Agency (CIA) World Factbook, to create a multilevel data set. Using stepwise multilevel modelling, we generated child-, family- and country-related factors that may have contributed to the anxiety levels of children with NDCs, their siblings if they had any, and their parents. All data were reported by parents.

**Results:**

Our results suggest that parental anxiety was best explained by family-related factors such as concerns about COVID-19 and illness. Children’s anxiety was best explained by child-related factors such as children’s concerns about loss of routine, family conflict, and safety in general, as well as concerns about COVID-19. In addition, anxiety levels were linked to the presence of pre-existing anxiety conditions for both children with NDCs and their parents.

**Conclusions:**

The present study shows that across the globe there was a raise in anxiety levels for both parents and their children with NDCs because of COVID-19 and that country-level factors had little or no impact on explaining differences in this increase, once family and child factors were considered. Our findings also highlight that certain groups of children with NDCs were at higher risk for anxiety than others and had specific concerns. Together, these results show that anxiety of families and their children with NDCs during the COVID-19 pandemic were predicted by very specific concerns and worries which inform the development of future toolkits and policy. Future studies should investigate how country factors can play a protective role during future crises.

The COVID-19 pandemic has considerably impacted people across the globe regarding finances, work, education, and health [1-3]. In the general population, the literature suggests increased anxiety, stress, depression, and emotional distress in many countries across the globe [4-8]. Epidemiological and psychological research documents the increased burden on mental health (especially anxiety levels) related to the COVID-19 pandemic and highlights its adverse effects on vulnerable groups, such as those with neurodevelopmental conditions (NDCs) [9-11] and their families [12].

The NDC umbrella includes a wide range of conditions and disabilities often linked to the functioning of the brain and neurological systems, such as attention-deficit / hyperactivity disorder (ADHD), autism, learning disabilities (LD), intellectual disabilities (ID), and developmental language disorder (DLD), as well as genetic and chromosomal conditions including Down syndrome (DS) and Williams syndrome (WS). Due to their cognitive, mental health, and medical profiles, children with NDCs often depend on support and care services and institutions, such as specialized schools, day care services, therapists, clinicians, or medical care, which differ in the amount and type of support provided [13]. The rapid escalation of COVID-19 from an epidemic to a pandemic interrupted numerous mechanisms and systems designed to protect public health and well-being across the globe [14], especially in relation to the cessation of mental health or support services. The effects of the COVID-19 pandemic have been shown to vary depending on country of residence [15], financial standing [16], and family context [17,18], and have significantly impacted the lives of families with a child with an NDC throughout the pandemic, although mainly during the first months [19].

While several studies have documented an increase in emotional and behavioural problems in response to the COVID-19 pandemic in children with NDCs as well as their parents and caregivers [9,20-24], most of these studies relied on small sample sizes, focused on populations within one country such as the UK, Italy, China and / or on one specific diagnostic group, e.g., autism, Down syndrome, or Prader-Willi syndrome [10,21,22,25]. Furthermore, no previous research has examined how factors at the country (e.g., restrictions, health care spending), family (e.g., parental concerns about finances, illness), and child (e.g., children’s concerns, health) level may explain the variability in parental and child anxiety in multiple groups of NDCs. Such an approach is critical to inform research, policy, and the design of support systems for future crises. Therefore, the present study aimed to quantify to what extent families and their children with NDCs were impacted by the COVID-19 pandemic and how their anxiety levels were mitigated or aggravated by country, family, and individual child factors such as their health or other individual differences or concerns.

More specifically, we aimed at examining the anxiety levels and concerns of families and their children with various types of NDCs in the early months of the pandemic and compared their anxiety levels pre-pandemic (retrospective), at the time of the first outbreak (retrospective), and as they completed the survey (from April to August 2020). These anxiety levels and concerns can be seen as indicators of the extent and appropriateness of government responses to the pandemic, an early reaction to how public health systems were coping with the crisis. Since the anxiety levels of the people were likely affected by national demographic characteristics or government policy (country level: e.g., number of deaths, fiscal measures, overall obesity levels, emergency health care investment), family context (family level: e.g., concerns about family safety, family conflict) and child context factors (child level: e.g., concerns about loss of routine, becoming ill themselves), we integrated data concerning how the pandemic evolved, government responses, and structural descriptors of countries from the European Centre for Disease Prevention and Control [26], the University of Oxford [27], and the CIA World Factbook [28] to a multilevel data set. This multilevel data set was used to assess which of these national characteristics, familial contexts, and child context factors predicted anxiety of parents and of their child with an NDC to understand how well the care and support system was perceived to be performing as a buffer to the economic, social, and political disruptions caused by the pandemic.

To summarize, this paper explores for the first time the extent to which the anxiety levels of parents and their children with an NDC can be explained by different levels of variance as we described above (country, family, and child levels). While we expected anxiety levels to increase at the beginning of the pandemic compared to pre-pandemic levels in both, the parents and their child with an NDC, we did not formulate specific hypotheses about which of these different levels (country, family, or child) would explain anxiety best due to the limited research on anxiety in families and their children with NDCs with a cross-country perspective taking into account all of these levels at the same time.

## METHODS

### Design

A cross-sectional online survey with a non-random sampling method was employed. This allowed us to gather sociodemographic and mental health perspectives of individuals with NDCs as well as of their families, including their typically developing siblings.

### Participants

Convenience sampling obtained 8043 participating families from seventy-eight countries (Section A, Table S1 in the **Online Supplementary Document**). After data cleaning, 6611 families from 70 countries remained, which included families with children with one of the most frequently diagnosed NDCs: autism, attention-deficit / hyperactivity disorder (ADHD), developmental language disorder (DLD), Down syndrome (DS), Williams syndrome (WS), and intellectual disability (ID). About one-third of the families also provided information about a typically developing sibling (TD). We decided to include the TD population in our analytical sample as previous research highlights that the mental health of siblings is often affected, either positively or negatively, by their sibling’s disabilities or neurodevelopmental conditions [29-31]. There were no constraints with respect to the age of the child with an NDC, so respondents could report about adults or children. For full sample descriptive statistics, see **Table 1**. Due to missingness in data, numbers vary per variable.

**Table 1:** Sample demographics and descriptive statistics

Variable	All (8043 / 10 200)		ADHD (417)		AUTISM (2796)		DLD (502)		DS (1330)		ID (1646)		WS (427)		TD (3083)	
	**Mean (count)**	**SD (%)**	**Mean (count)**	**SD (%)**	**Mean (count)**	**SD (%)**	**Mean (count)**	**SD (%)**	**Mean (count)**	**SD (%)**	**Mean (count)**	**SD (%)**	**Mean (count)**	**SD (%)**	**Mean (count)**	**SD (%)**
Age (P), years	42.93	10.03	41.35	8.82	41.81	8.8	38.78	9.25	46.13	10.34	43.01	11.21	45.89	10.9	43.16	9.72
Age (C), years	13.18	9.42	11.45	7.46	10.96	6.61	9.91	8.46	13.56	9.91	18.03	12.46	15.67	10.95	12.9	8.57
Anxiety now (P), 1-5	2.96	1.29	2.91	1.3	3.03	1.26	2.55	1.37	2.99	1.29	2.9	1.31	3.02	1.2	2.96	1.29
Anxiety now (P), 1-5	2.44	1.39	2.78	1.38	2.62	1.45	2	1.31	2	1.26	2.23	1.45	2.76	1.23	2.51	1.33
Sex (P)																
*Male*	2858	36%	115	28%	837	30%	186	37%	456	34%	825	50%	66	15%	1100	36%
*Female*	5146	64%	298	71%	1949	70%	308	61%	869	65%	809	49%	361	85%	1975	64%
Sex (C)																
*Male*	6122	60%	275	66%	2175	78%	315	63%	731	55%	1492	91%	237	56%	1492	48%
*Female*	3741	37%	131	31%	580	21%	176	35%	581	44%	1393	85%	187	44%	1393	45%
Relation to child																
*Father*	2516	31%	100	24%	787	28%	162	32%	400	30%	677	41%	63	15%	1004	33%
*Mother*	4641	58%	271	65%	1841	66%	290	58%	768	58%	633	38%	355	83%	1836	60%
*Other*	860	11%	44	11%	157	6%	46	9%	161	12%	328	20%	9	2%	238	8%
Education (P)																
*School leaving certificate*	2046	25%	115	28%	604	22%	167	33%	316	24%	498	30%	99	23%	771	25%
*University bachelor or equivalent*	2902	36%	151	36%	1041	37%	147	29%	500	38%	639	39%	120	28%	1106	36%
*University master or equivalent*	1392	17%	61	15%	616	22%	36	7%	263	20%	144	9%	100	23%	8592	19%
*Further vocational training*	859	11%	40	10%	306	11%	68	14%	141	11%	131	8%	82	19%	325	11%
*No formal qualification*	277	3%	18	4%	68	2%	47	9%	28	2%	91	6%	4	1%	71	2%
*Other*	529	7%	28	7%	149	5%	33	7%	79	6%	132	8%	19	4%	212	7%
Work situation (P)																
*Full time paid employment*	3316	41%	180	43%	1184	42%	196	39%	499	38%	726	44%	148	35%	1253	41%
*Part time paid employment*	1232	15%	58	14%	444	16%	45	9%	272	20%	160	10%	114	27%	520	17%
*Prime homemaker*	1725	21%	94	23%	618	22%	169	34%	243	18%	344	21%	70	16%	675	22%
*Retired*	678	8%	24	6%	147	5%	32	6%	165	12%	203	12%	38	9%	242	8%
*Student*	109	1%	8	2%	36	1%	12	2%	15	1%	20	1%	2	0%	38	1%
*Unemployed*	393	5%	22	5%	123	4%	30	6%	45	3%	102	6%	23	5%	125	4%
*Volunteer work*	83	1%	2	0%	33	1%	4	1%	15	1%	13	1%	6	1%	35	1%
*Other*	478	6%	27	6%	200	7%	12	2%	73	5%	67	4%	26	6%	190	6%
COVID-19 infection (P)																
*No*	6527	81%	319	76%	2244	80%	390	78%	1083	81%	1285	78%	373	87%	2787	90%
*Yes*	640	8%	38	9%	241	9%	26	5%	112	8%	103	6%	30	7%	287	9%
COVID-19 infection (C)																
*No*	8861	87%	343	82%	2401	86%	402	80%	1149	86%	1356	82%	395	93%	2815	91%
*Yes*	305	3%	15	4%	93	3%	12	2%	49	4%	34	2%	10	2%	92	3%
Anxiety disorder (P)																
*No*	4771	59%	223	53%	1594	57%	272	54%	845	64%	883	54%	300	70%	2223	72%
*Yes*	1433	18%	82	20%	553	20%	72	14%	180	14%	268	16%	37	9%	671	22%
Anxiety disorder (C)																
*No*	6112	60%	168	40%	1338	48%	288	57%	887	67%	780	47%	216	51%	2435	79%
*Yes*	1922	19%	117	28%	748	27%	46	9%	126	9%	323	20%	145	34%	417	14%
Living arrangement now (C)																
*At home with family*	9280	91%	384	92%	2638	94%	466	93%	1271	96%	1461	89%	413	97%	2647	86%
*In a group home*	199	2%	13	3%	61	2%	11	2%	26	2%	73	4%	5	1%	10	0%
*In a supported living setting*	109	1%	4	1%	43	2%	1	0%	15	1%	32	2%	8	2%	6	0%
*On his / her own*	110	1%	0	0%	22	1%	5	1%	0	0%	14	1%	1	0%	68	2%
*With a significant other*	157	2%	4	1%	8	0%	4	1%	2	0%	21	1%	0	0%	118	4%
*With roommate(s)*	66	1%	4	1%	8	0%	1	0%	6	0%	18	1%	0	0%	29	1%
School now (C)																
*At home with family*	7696	75%	391	94%	1989	71%	397	79%	1063	80%	1317	80%	320	75%	2329	76%
*Day care centre*	190	2%	7	2%	50	2%	1	0%	27	2%	39	2%	9	2%	57	2%
*Pre-school*	183	2%	7	2%	55	2%	16	3%	22	2%	12	1%	13	3%	58	2%
*School (mainstream)*	689	7%	79	19%	259	9%	30	6%	42	3%	35	2%	25	6%	219	7%
*School (special education)*	613	6%	17	4%	215	8%	25	5%	78	6%	74	4%	23	5%	181	6%
*Working in a job in the community*	4	0%	0	0%	0	0%	0	0%	0	0%	2	0%	1	0%	1	0%
*Working in a protected environment*	123	1%	3	1%	25	1%	2	0%	16	1%	27	2%	7	2%	43	1%
*Paid full or part time work*	86	1%	2	0%	23	1%	9	2%	6	0%	19	1%	4	1%	23	1%
*Other*	474	5%	11	3%	147	5%	6	1%	63	5%	85	5%	24	6%	138	4%
Environment now (C)																
*City / urban area*	5677	56%	240	58%	1582	57%	272	54%	711	53%	1019	62%	138	32%	1715	56%
*Town / suburban area*	2232	22%	88	21%	672	24%	97	19%	293	22%	267	16%	165	39%	650	21%
*Village / rural area*	2059	20%	76	18%	488	17%	114	23%	295	22%	300	18%	121	28%	665	22%
Additional diagnosis (NDC child)																
*No*	4622	57%	232	56%	1638	59%	362	72%	797	60%	791	48%	302	71%	.	.
*Yes*	3388	42%	179	43%	1147	41%	136	27%	532	40%	846	51%	125	29%	.	.
Intellectual disabilities (NDC child)																
*No*	2222	28%	214	51%	1147	41%	284	57%	105	8%	86	5%	8	2%	.	.
*Mild*	3983	50%	167	40%	1247	45%	190	38%	891	67%	832	51%	349	82%	.	.
*Severe*	1764	22%	25	6%	378	14%	22	4%	322	24%	711	43%	69	16%	.	.

SD – standard deviation, Perc – percentage, ADHD – attention-deficit / hyperactivity disorder, A – autism, DLD – developmental language disorder, DS – Down syndrome, ID – intellectual disability-not otherwise specified, TD – typically developing sibling, WS – Williams syndrome, NDC – neurodevelopmental conditions

### Materials

This study relies on a questionnaire that we developed at the beginning of the COVID-19 pandemic to assess how families and their children with NDCs were affected by the COVID-19 pandemic [32,33]. All materials, including the survey and related supplementary files can be accessed at: https://osf.io/5nkq9/. The first part of the questionnaire requested demographic information from a parent- or caregiver-respondent (for simplicity, we use the term “parent” here), including the primary diagnosis of their child with an NDC, presence or absence of intellectual disability, etc., see **Table 2** for a full list of included variables. Anxiety and specific concerns (e.g., about COVID-19, health, loss of institutional support) were assessed on a scale from 1 (not at all) to 5 (extremely) at three time points: retrospectively before the pandemic began, retrospectively at the start of the pandemic, and at the time of survey completion (between April and August 2020). Respondents reported anxiety and concerns for themselves, their child with an NDC, and optionally for their TD child (the complete survey, including further questions not considered here, can be found here [32,33]).

**Table 2:** Variables in stepwise regression, grouped by stepwise block (0-4), selection (yes or no), order at which the variable entered the model, and interactional (yes or no)

Variable	Stepwise block	Block N	Parental Anxiety Model selected	Parental Anxiety Model order	Parental Anxiety Model interaction	Child Anxiety Model selected	Child Anxiety Model order	Child Anxiety Model interaction
Diagnosis (C)	Base	0	Yes	1	Yes	Yes	1	Yes
Time	Base	0	Yes	1	Yes	Yes	1	Yes
Age (P)	Base	1	Yes	1	No	Yes	1	No
Sex (P)	Base	1	Yes	1	No	Yes	1	Yes
Age (C)	Base	1	Yes	1	Yes	Yes	1	Yes
Sex (C)	Base	1	Yes	1	No	Yes	1	No
Education (P)	Base	1	Yes	1	No	Yes	1	Yes
Relation to child	Base	1	Yes	1	Yes	Yes	1	No
Work situation (P)	Base	1	Yes	1	No	Yes	1	Yes
Anxiety disorder (C)	Base	1	Yes	1	No	Yes	1	Yes
Anxiety disorder (P)	Base	1	Yes	1	Yes	Yes	1	Yes
Days since the national peak	Base	1	Yes	1	Yes	Yes	1	Yes
Days since the pandemic	Base	1	Yes	1	Yes	Yes	1	Yes
Anxiety (P)	Family	3	Yes	1		Yes	5	Yes
Concerns own possible illness (P)*	Family	3	Yes	8	No	Yes	7	Yes
Anxiety (C)	Child	4	Yes	11	Yes	Yes	1	No
Public debt	Country	2	Yes	2	No	No		
Fiscal measures	Country	2	Yes	3	No	No		
Emergency health care investment	Country	2	Yes	4	No	No		
Concerns child approach (P)†	Family	3	Yes	9	No	No		
Concerns own safety (P)‡	Family	3	Yes	7	No	No		
Concerns C-19 (P)§	Family	3	Yes	6	Yes	No		
Concerns child health (P)‖	Family	3	Yes	8	No	No		
Concerns own illness (P)¶	Family	3	Yes	5	Yes	No		
Airports	Country	2	No			Yes	2	No
Cancelation of public events	Country	2	No			Yes	4	No
Obesity	Country	2	No			Yes	3	No
Concerns child ability (P)**	Family	3	No			Yes	6	Yes
Concerns family conflict (P)††	Family	3	No			Yes	8	Yes
Concerns child motivation (P)‡‡	Family	3	No			Yes	9	No
Concerns COVID-19 (C)§§	Child	4	No			Yes	11	Yes
Concerns family conflict (C)‖‖	Child	4	No			Yes	14	No
Concerns illness (C)¶¶	Child	4	No			Yes	10	Yes
Concerns routine (C)***	Child	4	No			Yes	13	Yes
Concerns safety (C)†††	Child	4	No			Yes	12	Yes
Country area	Country	2	No			No		
Budget surplus	Country	2	No			No		
School closing at peak	Country	2	No			No		
School closing max	Country	2	No			No		
Workplace closing at peak	Country	2	No			No		
Work closing max	Country	2	No			No		
Cancelation of public events	Country	2	No			No		
Restrictions on gathering at peak	Country	2	No			No		
Restrictions on gathering max	Country	2	No			No		
Close public transportation at peak	Country	2	No			No		
Closing public transportation max	Country	2	No			No		
Stay at home at peak	Country	2	No			No		
Stay at home max	Country	2	No			No		
Restrictions on international travel at peak	Country	2	No			No		
Restrictions on international travel max	Country	2	No			No		
Cellphone users	Country	2	No			No		
Containment health index at peak	Country	2	No			No		
Containment health index max	Country	2	No			No		
Death rate	Country	2	No			No		
Death rate total	Country	2	No			No		
Deaths per month	Country	2	No			No		
Deaths per month total	Country	2	No			No		
Income support at peak	Country	2	No			No		
Income support max	Country	2	No			No		
Contract relief at peak	Country	2	No			No		
Contract relief max	Country	2	No			No		
Fiscal measures max	Country	2	No			No		
International support max	Country	2	No			No		
Economic support index at peak	Country	2	No			No		
Economic support index max	Country	2	No			No		
Education	Country	2	No			No		
Gross domestic product per capita	Country	2	No			No		
Government response index at peak	Country	2	No			No		
Government response max	Country	2	No			No		
Public information campaigns at peak	Country	2	No			No		
Public information campaigns max	Country	2	No			No		
Testing policy at peak	Country	2	No			No		
Testing policy max	Country	2	No			No		
Contact tracing max	Country	2	No			No		
Emergency health care investment max	Country	2	No			No		
Investment in vaccines max	Country	2	No			No		
Inflation	Country	2	No			No		
Internet users	Country	2	No			No		
Median age	Country	2	No			No		
Net migration	Country	2	No			No		
Population	Country	2	No			No		
Railways	Country	2	No			No		
Roadways	Country	2	No			No		
Stringency index at peak	Country	2	No			No		
Stringency index max	Country	2	No			No		
Country taxes	Country	2	No			No		
Telephone users	Country	2	No			No		
Unemployment	Country	2	No			No		
Youth unemployment	Country	2	No			No		
School closing now	Country	2	No			No		
Workplace closing now	Country	2	No			No		
Cancelation of public events now	Country	2	No			No		
Restrictions on gatherings now	Country	2	No			No		
Close of public transportation now	Country	2	No			No		
Stay at home now	Country	2	No			No		
Restrictions on international movement now	Country	2	No			No		
International travel controls now	Country	2	No			No		
Containment health index now	Country	2	No			No		
Deaths now	Country	2	No			No		
Deaths per month now	Country	2	No			No		
Income support now	Country	2	No			No		
Contract relief now	Country	2	No			No		
International support now	Country	2	No			No		
Economic support index now	Country	2	No			No		
Government response now	Country	2	No			No		
Public information campaigns now	Country	2	No			No		
Testing policy now	Country	2	No			No		
Contact tracing now	Country	2	No			No		
Investment in vaccines now	Country	2	No			No		
Stringency index now	Country	2	No			No		
Current government guidance	Family	3	No			No		
Family infected with COVID-19	Family	3	No			No		
First hear about COVID0-19	Family	3	No			No		
Following gov. advice	Family	3	No			No		
Concerns family’s safety (P)‡‡‡	Family	3	No			No		
Self-isolation	Family	3	No			No		
Speaking to expert desire	Family	3	No			No		
Schools closed	Family	3	No			No		
Concerns self-isolation (P)	Family	3	No			No		
Self-isolation pre-gov. guidance	Family	3	No			No		
Self-isolation (P)§§§	Family	3	No			No		
Expert discussion about child (P)	Family	3	No			No		
Concerns family safety post-social distancing announcement (P)	Family	3	No			No		
Concerns child boredom (P)‖‖‖	Family	3	No			No		
Concerns child illness (P)¶¶¶	Family	3	No			No		
Concerns finance (P)****	Family	3	No			No		
Concerns child institution (P)††††	Family	3	No			No		
Concerns child social (P)‡‡‡‡	Family	3	No			No		
Concerns work balance (P)§§§§	Family	3	No			No		
Occupation before (C)	Child	4	No			No		
Urbanity before (C)	Child	4	No			No		
Urbanity now (C)	Child	4	No			No		
Occupation now (C)	Child	4	No			No		
Fear communication (C)	Child	4	No			No		
Health index (C)	Child	4	No			No		
ID (C)	Child	4	No			No		
Residence before (C)	Child	4	No			No		
Residence now (C)	Child	4	No			No		
Medical issues (C)	Child	4	No			No		
COVID-19 infection (C)	Child	4	No			No		
COVID-19 awareness (C)	Child	4	No			No		
Concerns approach (C)‖‖‖‖	Child	4	No			No		
Concerns boredom (C)¶¶¶¶	Child	4	No			No		
Concerns finances (C)*****	Child	4	No			No		
Concerns friends (C)†††††	Child	4	No			No		
Concerns own illness (C)‡‡‡‡‡	Child	4	No			No		
Concerns health (C)§§§§§	Child	4	No			No		
Concerns institution (C)‖‖‖‖‖	Child	4	No			No		
Concerns others illness (C)¶¶¶¶¶	Child	4	No			No		
Typically developing sibling health problems	Child	4	No			No		

*How concerned were / are you about the possibility that you will get ill?

†How concerned were / are you that your child is not able to approach others?

‡How concerned were / are you about your child’s safety with respect to COVID-19?

§How concerned were / are you about COVID-19?

‖How concerned were / are you about your child’s health?

¶How concerned were / are you about illness in general?

**How concerned were / are you about your child’s ability to cope with changes in his / her routine?

††How concerned were / are you about family conflict (fights, aggression)?

‡‡How concerned were / are you about your ability to keep your child with NDCs entertained and motivated?

§§How concerned was / is your child about COVID-19?

‖‖How concerned was / is your child about family conflict?

¶¶How concerned was / is your child about illness in general?

***How concerned was / is your child about his or her loss of routine?

†††How concerned was / is your child about the family’s safety with respect to COVID-19?

‡‡‡How concerned did you feel at the tie for your family’s safety?

§§§Did your relevant government (city, state or country) suggest that you and your family should self-isolate? Self-isolation means stay at home (Yes / No answers)

‖‖‖How concerned were / are you about your child becoming bored?

¶¶¶How concerned were / are you about the possibility that your child will get ill?

****How concerned were / are you about your financial / economic situation?

††††How concerned were / are you about the loss of institutional (eg, school, workplace) support for your child including interventions (language therapists, psychologists etc.)?

‡‡‡‡How concerned were / are you about the fact that your child has fewer occasions for social contact and interaction?

§§§§How concerned were / are you about work / childcare balance related to looking after a child with NDCs?

‖‖‖‖How concerned was / is your child about not being able to approach others?

¶¶¶¶How concerned was / is your child about boredom?

*****How concerned was / is your child about his financial / economic situation?

†††††How concerned was / is your child about not being able to meet peers and friends?

‡‡‡‡‡How concerned was / is your child about the possibility that he / she will get ill?

§§§§§How concerned was / is your child about his / her own health?

‖‖‖‖‖How concerned was / is your child about the loss of institutional (eg, school, workplace) support for your child including interventions (language therapist, psychologist etc.)?

¶¶¶¶¶How concerned was / is your child about the possibility that others will get ill?

### Procedure and ethics

The link to the online questionnaire was distributed via the networks of participating researchers worldwide in more than 24 countries and international family associations. The questionnaire was available in 16 languages (see limitations) [32,33]. Recruitment flyers were sent to associations and charities to invite parents and caregivers to report about their child with an NDC. Ethical approval for this anonymous questionnaire was obtained by the institutional review board of UniDistance Suisse.

### Data analysis

The primary outcomes were self-reported (parent) anxiety and parent-reported anxiety of the child with an NDC (continuous variable) and of the typically developing (TD) child, which we analysed using stepwise multilevel linear regression. This model allowed us to treat individual families and countries as nested sources of random / sampling variance while handling predictors of fixed variance in the usual fashion (changing them in every block). Four blocks of fixed effects were added progressively to the model, consisting of (i) design or confirmatory variables (e.g., NDC group) in the base model, (ii) country variables (e.g., government response to COVID-19 [25]), (iii) family variables (e.g., family situation, parent concerns about COVID-19; concerns about their health and concerns about their child with an NDC), and (iiii) child variables (e.g., the child with NDC’s reported concerns about COVID-19; and concerns about motivation to name a few). As such, blocks (ii)-(iiii) reflected different levels of sources of anxiety from macro (country) to micro-level (child factors). By adding and eliminating variables within each block, we obtained a parsimonious model containing only the most important predictors for parental anxiety and anxiety of the child with an NDC. In selecting important effects, we focused on how time and the different levels of factors (family, country, and child) modified group dynamics in anxiety and whether they were mitigating or aggravating influences. Model output was quantified as marginal- and conditional-R^2^ for goodness-of-fit, F- and *t* tests for the inferential significance of predictors, and standardized regression differences as measures of effect. A full description of the data analysis procedure, including the list of variables in each regression block as well as how the missing data were handled, can be found in the **Online Supplementary Document**. The stepwise model sequence and fit statistics are also presented in section A, Table S2 in the **Online Supplementary Document**. In addition, Type II ANOVA breakdown for the final multilevel regression models are presented in section A, Table S3 in the **Online Supplementary Document**.

## RESULTS

Our data show that the mean age for the participating parents was 42.93 years and 13.18 years for their child. The mean level of anxiety for the participating parent was 2.96 and 2.44 for their child (on a scale of 1 to 5). 17% of the parents had a university degree, and among the responding parents, the majority (58%) were mothers of children with an NDC.

### Parent anxiety

**Figure 1:**
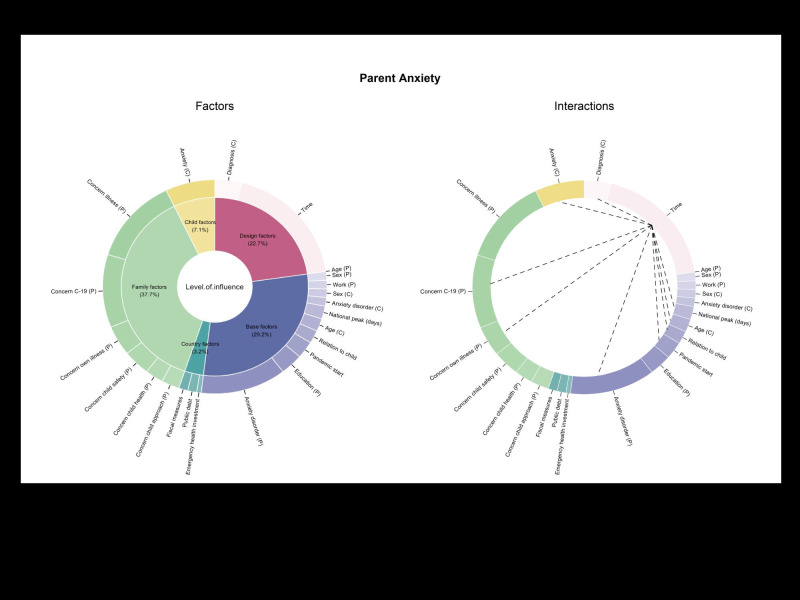
Breakdown of model effects and interactions for Parent Anxiety Model. Black lines represent significant Time interactions.

The final model explained 60% of the marginal variation (R^2^marg = 0.601) in parent anxiety. The confirmatory predictors explained 21.9%, with country-, family-, and child-related variables incrementally adding 0.5%, 36.8% and 0.9%, respectively. This suggested that parent anxiety was best explained by family-related factors, as shown in **Figure 1** for a breakdown of model effects and Table S3A in the **Online Supplementary Document** for the detailed Type II ANOVA of the final multilevel regression for parent anxiety.

**Figure 2:**
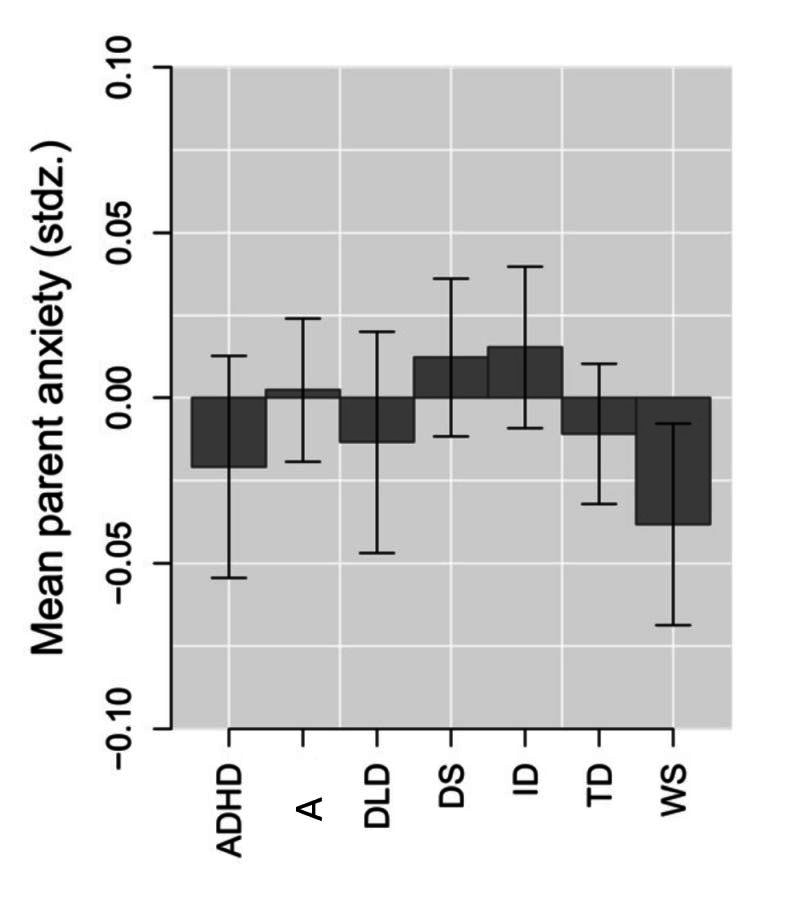
Standardised Mean Parent Anxiety per NDCs. The lower the mean the greater the anxiety. Stdz – standardized, NDCs – neurodevelopmental conditions, ADHD – attention-deficit / hyperactivity disorder, A – autism, DLD – developmental language disorder, DS – Down syndrome, ID – intellectual disability-not otherwise specified, TD – typically developing sibling, WS – Williams syndrome

The final model did not reveal a main effect of diagnosis group, *F*(6,11368.0) = 1.15, *P* = 0.3327, βz = 0.060, suggesting that parents experienced on average the same anxiety regardless of the NDC of their child (**Figure 2**).

**Figure 3:**
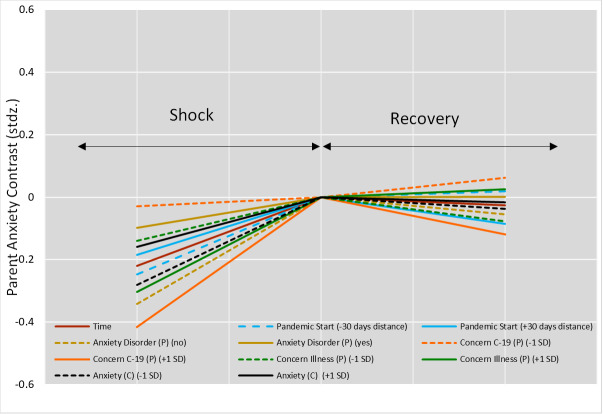
Parent Anxiety Time Main Effect & Interactions with the Shock vs Recovery Effect. Peak is anchored to 0 to show the relative changes with respect to the U-Shape Turn. (**C**) refers to variables concerning the child, (**P**) refers to variables concerning the parent or caregiver. +1SD refers to a positive standard deviation away from the mean, -1SD refers to a negative standard deviation away from the mean. All variables are explained in **Table 2**.

The final model revealed a main effect of time; *F*(2,17.654) = 160.33, *P* < 0.0001, βz = 0.286, such that average parental anxiety followed an inverted U-shape profile across the three time points. This profile consisted of a “shock”, a “peak”, and a “recovery”, with the shock represented by the large increase in average anxiety from before to the start of the COVID-19 pandemic; *t*(22.45) = -11.24, *P* = 0.000, βz = -.38), and the recovery represented by a decrease in average anxiety from the start of the pandemic to the now moment (ie, the moment the participants responded to the questionnaire), *t*(17.63) = 2.15, *P* = 0.045, βz = 0.057). This main effect was modified by nine interaction effects, some of which changed shock, recovery or both, as shown in line for Time in **Figure 3**. A higher-than-average concern about COVID-19 magnified the shock effect and speeded recovery, vs the average time trend, as shown in line concerns C-19 (P) in **Figure 3**. By contrast, having lower-than-average concerns about COVID-19 flattened the U-shaped time trend compared to the average, as shown in dashed concerns C-19 (P) in **Figure 3**.

**Figure 4:**
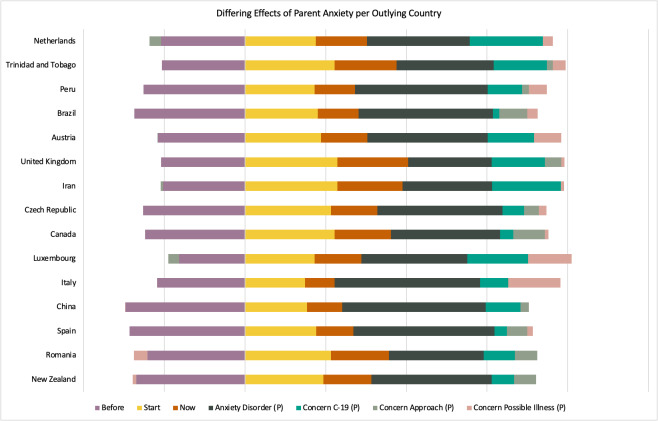
Differing effects of parent anxiety per outlying country. (**P**) refers to variables concerning the parent or caregiver.

Although some country factors such as country public debt, government fiscal measures in response to COVID-19 now, and emergency investment health care spending at the time of the participant completed the survey were predictive of parent anxiety and selected during stepwise model building, these factors were all non-significant in the final model (section A, Table S3 in the **Online Supplementary Document**), suggesting that these differences were better explained by family- and child-related factors. In general, the country’s structural characteristics did not affect parental anxiety, nor did the national government’s response to the COVID-19 pandemic. Parental anxiety was strongly predicted by specific health-related concerns, including the parent’s possibility of becoming ill, as presented with green line in **Figure 3**, or COVID-19 specifically, as presented with orange line in **Figure 3**. Parental anxiety was also strongly and solely predicted by the level of anxiety of their child with an NDC, in that parents who were anxious themselves tended to report higher anxiety in their children. Although the final model showed that the factors discussed above impacted parental anxiety, it is important to note that degree to which these four factors influenced anxiety differed between countries as reflected in the final model’s random effect structure. **Figure 4** shows which countries were outlying for the differing effects. For example, parent anxiety levels in the Netherlands were best explained by parental concerns about COVID-19 and least explained by their concerns about Possible Illness compared to Luxembourg where concerns about Possible Illness had a stronger impact. It is important to note that these countries were not outliers specifically due to their sample size.

### Anxiety reported for children with NDCs and their TD siblings

**Figure 5:**
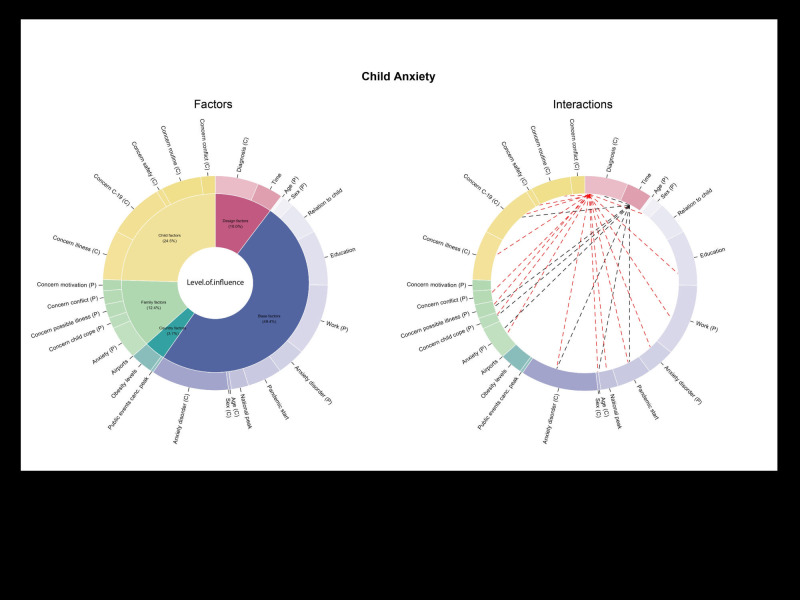
Breakdown of model effects and interactions for Child Anxiety Model. Black lines represent significant Time interactions, and red lines represent significant Diagnosis interactions.

The final model explained 57.6% of R^2^marginal variation in levels of anxiety for children with NDCs and their TD siblings. The confirmatory predictors explained 23%, with country-, family-, and child-related variables incrementally adding 2.8%, 9.7% and 21.2%, respectively. This suggested that levels of anxiety for children were best explained by the child factors in addition to the base factors. For a breakdown of model effects see **Figure 5** and section A, Table S3B in the **Online Supplementary Document** for the detailed Type II ANOVA of the final multilevel regression for child anxiety.

**Figure 6:**
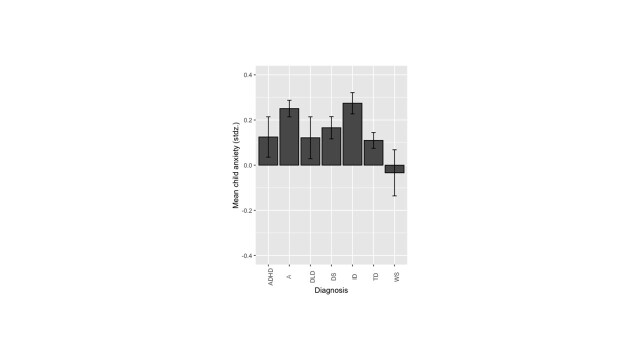
Standardised mean child anxiety per NDCs. The lower the mean the greater the anxiety. Stdz – standardized, NDCs – neurodevelopmental conditions, ADHD – attention-deficit / hyperactivity disorder, A – autism, DLD – developmental language disorder, DS – Down syndrome, ID – intellectual disability-not otherwise specified, TD – typically developing sibling, WS - Williams syndrome

The final model revealed a main effect of the diagnosis group, *F*(6,13510.4) = 9.90, *P* < 0.0001, βz = -.154, suggesting that parents reported levels of child anxiety depended on the NDC of their child (**Figure 6**). This figure shows also that children with WS had the highest anxiety levels. This main effect was modified by 11 interaction effects, which reflected that some variables were more important in predicting anxiety for specific NDC groups with a notable interaction on pre-existing anxiety disorder interacting with the diagnosis; *F*(6,13533.2) = 4.61, *P* < 0.0001, βz = 0.066.

**Figure 7:**
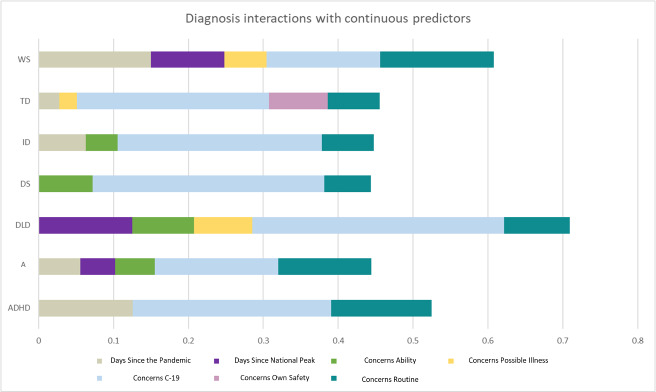
Diagnosis interactions with continuous predictors. WS – Williams syndrome, TD – typically developing sibling, ID – intellectual disability-not otherwise specified, DS – Down syndrome, A – autism, ADHD – attention-deficit / hyperactivity disorder, DLD – developmental language disorder.

Temporal proximity to the start of the pandemic and days since national peak predicted most strongly child anxiety for individuals with WS, whereas this had no impact on the DS and DLD groups. Likewise, whereas concerns about COVID-19 strongly predicted child anxiety in all groups, only for WS, TD, and DLD did child concerns about illness in general play an additional role. For all groups, child concerns about the loss of routine were a strong predictor, but it was the most relevant for individuals with WS, autism, and ADHD (**Figure 7**).

**Figure 8:**
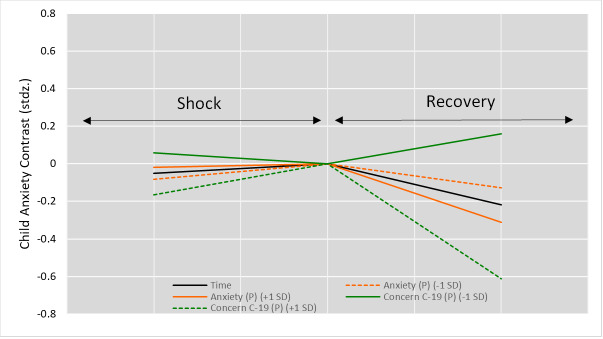
Child Anxiety Time Main Effect & Interactions with the Shock vs. Recovery Effect. Stdz – standardised, (**P**) refers to variables concerning the parent or caregiver. +1SD refers to a positive standard deviation away from the mean, -1SD refers to a negative standard deviation away from the mean. All variables are explained in **Table 2**.

The final model revealed a main effect of time *F*(2,17.654) = 160.33, *P* < 0.0001, βz = 0.286, such that average child anxiety followed an inverted U-shape profile across the three time points. Similarly to parental anxiety, this profile consisted of a “shock”, a “peak”, and a “recovery”, with the shock the large increase in average anxiety from before to the start of COVID-19 *t*(82.5) = -3.73, *P* = 0.0000, βz = -.069, and the recovery a decrease in average anxiety from the start to the now moment *t*(37.8) = -.82, *P* = 0.41, βz = -.016. This main effect was modified by eight interaction effects, some of which changed shock, recovery, or both, as shown in **Figure 8**.

**Figure 9:**
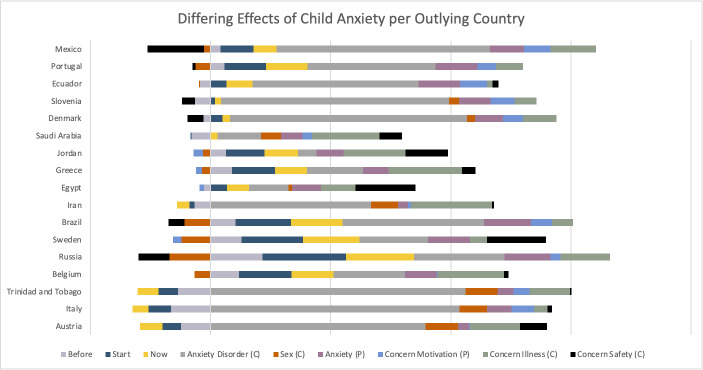
Differing effects of child anxiety per outlying country. (**C**) refers to variables concerning the child, (**P**) refers to variables concerning the parent or caregiver.

A higher-than-average concern about COVID-19 magnified the shock effect and sped up recovery vs the average time trend. By contrast, having lower-than-average concerns about COVID-19 flattened the U-shaped time trend compared to the average. As for the country effects, country public debt, government fiscal measures in response to COVID-19 up until the now time point, and emergency investment in health care spending now were selected during stepwise model building as predictive of child anxiety, although all were non-significant in the final modelling, suggesting these differences were better explained by family- and child-related factor differences. Finally, several effects from the four levels of influence also differed between countries, as reflected in the final model’s random effect structure. **Figure 9** shows which countries were outlying with respect to the differing effects. For example, children’s anxiety was best explained by children’s anxiety disorder in Mexico compared to Greece where children’s concerns about Illness had a bigger effect. Similarly, the cause of these countries being outliers was not specifically due to sample size.

## DISCUSSION

In this international study, we examined the anxiety and concerns of children with NDCs and their families. We assessed which factors relating to the country and government policy, family context, or child contexts were more likely to be related to increased anxiety using a multilevel approach. By adopting a cross-country perspective, this study adds to the mounting evidence of widespread increased stress and mental health concerns, specifically anxiety, in children with NDCs and their caregivers [24]. Parental anxiety was best explained first by family- and child-related predictors and then by country-related predictors. Similarly, children’s anxiety was explained best by child- and family-related predictors followed by country-related predictors. Anxiety levels increased strongly at the beginning of the pandemic in both parents and children which coincided with the first peak of the pandemic in the respective countries (with the peak of the pandemic being defined as the maximum number of deaths between April to August 2020) which aligns with previous research [2,5,9,10,20,21,34,35]. Over time, parental anxiety decreased slightly as the time distance from the first peak increased but it did not return to the perceived pre-pandemic level, indicating chronic increased stress in these families due to the pandemic and pandemic-related issues. However, this was not the case for child anxiety as a decrease in average anxiety from the start of the pandemic to the now moment was revealed in the final model.

Anxiety in both parents and children was linked to pre-existing anxiety conditions, so a pre-existing anxiety condition affected parental anxiety which also impacted the anxiety of their children and vice versa. In addition, we observed that various concerns affected anxiety strongly in both parents and their children with NDCs. Parents were mostly concerned about their children with NDCs not being able to approach others, COVID-19, and possibly getting ill. These concerns likely reflected parents’ fear that their children were at risk of contracting a serious form of COVID-19 and the impact that it would have on their child’s long-term health (e.g., children with WS have higher chances of experiencing severe medical issues [36,37]). In relation to child factors, parental anxiety was best explained through concerns about their children’s motivation, illness, and safety. Nevertheless, it is crucial to analyse country, family, and child characteristics, in addition to concerns that impacted the anxiety of parents and children the most. To date, no research has investigated the effects of COVID-19 using a global sample and employing cross-country models, hence it is difficult to comment on such findings.

Surprisingly, none of the government-level policy measures were significant factors predicting parental anxiety in the final model, which indicates that country-related contexts such as the public health system of a country did not have a direct impact on parental anxiety. However, when we added additional predictors to the analysis, variables other than government-related factors became significant. In the final multilevel analysis model, parental concerns related to their health, their children’s health, and their children’s lack of opportunity to get physically close to others due to anti-transmission measures (e.g., two-meter distance, social distancing) were better predictors of parental anxiety. In sum, health-related worries and concerns about limited opportunities for social interactions due to anti-transmission measures increased parental anxiety. The lack of social contact seems to be one of the main worries for families with a child with an NDC, likely related to concerns about their social development [38]. Interestingly, parental anxiety was not impacted by their child’s condition (group of NDC), showing that parents from different groups had similar anxiety levels. However, there were some differences between countries in how much family-related factors and concerns contributed to the parents’ anxiety. In sum, parental anxiety is mostly mediated by personal concerns and family factors within the context of a country, but not by country level factors per se.

When examining children’s anxiety levels, we see a similar picture. The government policies and family practices, which can be seen as vital components of the global public health system in protecting vulnerable children, did not impact immediately on children's anxiety once family and child factors were taken into account. Instead, children’s anxiety was specifically related to base factors such as their sex and age to name a few. In addition, child factors played a key role in explaining anxiety in children with NDCs such as concerns about COVID-19. A detailed overview is presented in **Figure 5**. In addition, the analysis revealed how a child’s diagnostic group made a difference in how they coped with the onset of the pandemic, indicating that syndrome-specific vulnerability or proneness to anxiety impacted individual and group anxiety levels. In line with previous research [39,40], we identified the highest anxiety levels in children with WS. As revealed in several interactions within the NDC group, certain variables, including anxiety disorder and the child’s and parent’s specific concerns (e.g., about COVID-19, illness in general), seemed to affect the child’s anxiety in several diagnostic groups (section A, Table S3 in the **Online Supplementary Document**). Moreover, interactions with the diagnostic group were revealed (e.g., concerns about changes in their routine) which is in line with the generally elevated need of children with NDCs for consistency [41,42]. This finding indicates that the sudden changes to the daily structures of children with NDCs during the COVID-19 pandemic, due to the closure of schools and institutions, the discontinuation of mental health care services, as well as the necessary familial reorganization, greatly impacted these children and their families, independently of the country they lived in.

While this study's strength is undoubtedly the large sample size and the number of countries included, several limitations need to be mentioned: The study (a) used self-reported NDC diagnoses; (b) relied on parent-reported anxiety for children; (c) did not use standardized tools to assess anxiety and specific concerns due to methodological constraints (e.g., the total length of the questionnaire, the availability of these tools in all languages, the lack of pilot data and validation of the tools); (d) the translation of the survey in other languages was not conducted by professionals but by the collaborators, (e) the study involved retrospective reporting about the course of the anxiety rather than contemporaneous rating. However, new research shows that humans can operationalize a scale for their emotions, hence the measure of anxiety through a single scale should still give us insights into their anxiety and stresses [43,44]. Nevertheless, the way anxiety has been assessed does not allow us to draw conclusions concerning the extent to which the anxiety reached clinical relevance. Despite these limitations, we believe that the current study can contribute to understanding the factors that influenced families with a child with an NDC during the first months of the COVID-19 pandemic, as previous research for single countries showed [9,21,45,46], and how country-specific, family, and child factors contributed to parent and child anxiety.

### Implications

Whereas family factors such as concerns about COVID-19, the child’s safety, and the child’s health, to name a few examples, explained parental anxiety best, governmental measures and policies (**Table 2**) did not directly impact parental anxiety once some specific fixed-factor predictors were added in the final model. Similarly, for children with NDCs, country-level factors did not explain variance in anxiety once family and child-level factors were considered. Child factors such as concerns about COVID-19, family conflict, and loss of routine explained children with NDCs’ anxiety and showed that despite there being group-specific concerns and anxiety levels, the lack of routine was a significant contributor to all children’s anxiety. Taking these findings together, this study provides insight for future policy recommendations and is also informative for developing interventions and toolkits to help parents (who were more affected by anxiety), and children cope with future crises. For instance, toolkits that the public health system could provide to support families with children with NDCs through events such as a pandemic should emphasize the importance of re-establishing a new family routine, or to help children as well as their parents to regulate their anxiety, as other researchers have argued in the literature [10,47]. In addition, the findings show that parents and children had very specific concerns that are context specific and should be addressed in future crises. However, future studies should examine this context-specific nature and whether the fact that we did not identify an influence of country-level factors (e.g., public debt; fiscal measures and emergency health care investment) was because parents did not feel supported or because there was another common factor across different countries (e.g., lack of sustainable support through the pandemic).

## Additional material


Online Supplementary Document
