# Supplementary material for: Anxiety, concerns and COVID-19: Cross-country perspectives from families and individuals with neurodevelopmental conditions
Source: J Glob Health. 2023 Jul 28;13:04081. doi: 10.7189/jogh.13.04081 (PMC10373111; doi:10.7189/jogh.13.04081)
Supplement: Online Supplementary Document [file jogh-13-04081-s001.pdf]

Online Supplementary Documents

Supplementary Materials A

Table S1. Sample sizes by country. Number of unique families, children, and diagnosis groups.

Country	Families	Children	ADHD	AUTISM	DLD	DS	ID	WS	TD
Algeria	1	1	0	1	0	0	0	0	0
Angola	1	1	0	0	1	0	0	0	0
Argentina	4	5	0	2	1	0	0	1	1
Australia	90	120	3	68	0	4	9	0	36
Austria	46	60	1	25	0	10	4	1	19
Bahrain	4	5	0	1	0	0	2	0	2
Belarus	5	8	0	1	0	1	3	0	3
Belgium	47	64	1	2	0	41	1	1	18
Bolivia	3	4	0	2	1	0	0	0	1
Brazil	30	39	1	22	0	1	1	0	14
Bulgaria	1	1	1	0	0	0	0	0	0
Canada	26	36	0	12	0	2	1	5	16
Central African Republic	1	1	0	0	0	0	1	0	0
Chile	1	1	0	1	0	0	0	0	0
China	536	627	12	193	165	30	122	0	105
Colombia	2	2	0	2	0	0	0	0	0
Congo, Republic of the...	1	1	0	1	0	0	0	0	0
Cyprus	3	4	0	3	0	0	0	0	1
Czech Republic	86	109	18	28	6	9	12	7	29
Dem. Rep. Korea	1	1	0	1	0	0	0	0	0
Denmark	2	3	0	1	0	0	0	1	1
Ecuador	42	55	1	32	1	6	0	0	15
Egypt	5	6	0	2	0	0	1	1	2
El Salvador	1	1	0	1	0	0	0	0	0
Ethiopia	1	1	0	1	0	0	0	0	0
France	97	125	2	12	1	13	6	22	69
Georgia	1	1	0	0	0	0	0	1	0
Germany	800	1026	26	196	10	179	140	101	374
Greece	10	14	2	5	0	1	1	1	4
Guatemala	1	1	0	1	0	0	0	0	0
India	23	28	7	13	0	1	2	0	5
Iran	112	140	1	103	1	3	3	0	29
Ireland	52	76	0	26	0	8	5	10	27
Israel	3	3	0	2	0	0	0	1	0
Italy	112	157	26	40	4	9	5	24	49
Jordan	4	5	0	1	0	0	1	0	3
Kazakhstan	2	2	0	2	0	0	0	0	0
Kenya	1	1	0	0	0	0	0	0	1

Kuwait	1	1	0	1	0	0	0	0	0
Lebanon	1	2	0	0	0	0	0	1	1
Libyan Arab Jamahiriya	1	1	0	0	0	0	1	0	0
Lithuania	1	1	0	1	0	0	0	0	0
Luxembourg	11	15	0	2	0	6	0	1	6
Mexico	10	13	0	9	0	0	1	0	3
Namibia	3	4	0	2	0	0	1	0	1
Netherlands	67	92	1	3	0	60	0	2	26
New Zealand	15	18	2	3	0	8	0	0	5
Nicaragua	1	1	0	1	0	0	0	0	0
Nigeria	2	2	0	0	0	2	0	0	0
Norway	1	2	0	0	0	0	0	1	1
Peru	9	10	0	8	0	1	0	0	1
Poland	2	2	0	1	0	0	0	1	0
Portugal	189	233	32	95	13	16	14	2	61
Qatar	3	3	0	3	0	0	0	0	0
Romania	79	102	5	51	5	7	5	0	29
Russia	196	238	6	105	8	15	19	16	69
Saint Kitts and Nevis	1	1	0	0	0	0	0	0	1
Saudi Arabia	3749	4698	195	994	253	593	1194	10	1459
Serbia	1	1	0	1	0	0	0	0	0
Singapore	2	2	0	2	0	0	0	0	0
Slovakia	42	60	2	18	2	3	1	16	18
Slovenia	1	2	0	1	0	0	0	0	1
South Africa	4	5	0	2	0	2	0	0	1
Spain	65	89	1	15	2	23	6	17	25
Swaziland	1	1	0	0	0	1	0	0	0
Sweden	97	129	10	56	2	9	10	3	39
Switzerland	348	477	12	161	3	96	25	15	165
Tanzania	1	2	0	0	0	1	0	0	1
Trinidad and Tobago	5	6	0	4	0	1	0	0	1
Tunisia	1	1	0	0	0	0	0	0	1
Turkey	1	1	1	0	0	0	0	0	0
Uganda	1	1	0	0	0	0	1	0	0
United Arab Emirates	7	10	1	2	2	1	1	0	3
United Kingdom	444	557	29	182	12	122	21	56	135
United States	522	682	18	270	9	45	25	109	206
Total	8043	10200	417	2796	502	1330	1645	427	3083

Table S2. Stepwise model sequence and fit statistics.

**Outcome**	**Model**	**Fixed effects**	**Random effects**	R^2_{marg}	**AIC**	N_{total}	N_{family}	$N_{country}$
	Null	0	2	0.000	67001.9	25674	6611	66

Parent anxiety	+ Design	3	3	0.123	61934.9	25674	6611	66
	+ Base aspects	19	6	0.219	49577.8	21142	5414	61
	+ Country aspects	22	5	0.224	49234.3	20986	5379	60
	+ Family aspects	30	7	0.592	35276.8	20034	5223	59
	+ Child aspects	32	8	0.601	34395.1	19673	5146	59
Child anxiety	Null	0	2	0.000	58458.6	23272	5951	63
	+ Design	3	4	0.055	56326.2	23272	5951	63
	+ Base aspects	25	5	0.230	47514.7	20866	5324	61
	+ Country aspects	28	5	0.258	47204.8	20713	5290	60
	+ Family aspects	40	7	0.355	42830.7	19856	5148	60
	+ Child aspects	50	9	0.567	31913.2	19124	5008	59

Note: R^2_{marg}: Marginal R^2. AIC: Akaike Information Criterion.

Table S3A. Type II ANOVA breakdown of the final multilevel regression for Parent Anxiety. Max. Beta indicates the maximum standardized contrast among pairwise differences. * = $p <$ 0.005

**Effect**	**Block**	**Max. Beta**	***F***	**DF**	***p***	
Age (P)	Base	0.000	0.00	(1,5709.1)	0.9973	
Sex (P)	Base	0.022	1.49	(1,5269.9)	0.2216	
Relation to child	Base	-0.029	0.00	(2,5382.7)	0.9989	
Education	Base	0.054	1.70	(5,4770.4)	0.1310	
Work Situation (P)	Base	0.022	0.24	(7,4893.0)	0.9757	
Anxiety Disorder (P)	Base	0.194	161.53	(1,14.9)	0.0000	*
Days Since Pandemic	Base	0.040	0.87	(1,62.2)	0.3555	
Days Since National Peak	Base	-0.027	3.21	(1,80.1)	0.0768	
Diagnosis (C)	Base	-0.060	1.15	(6,11368.0)	0.3327	
Age (C)	Base	-0.027	5.07	(1,8167.6)	0.0244	
Sex (C)	Base	0.022	0.99	(1,13398.2)	0.3190	
Anxiety Disorder (C)	Base	-0.022	14.02	(1,11893.7)	0.0002	*
Time	Base	0.286	160.33	(2,17.6)	0.0000	*
Public Debt	Country	0.016	1.16	(1,101.0)	0.2833	
Fiscal Measures	Country	0.018	1.00	(1,3658.5)	0.3168	
Emergency Health Investment	Country	-0.014	0.63	(1,4084.6)	0.4290	
Concern Illness (P)	Family	0.198	1295.55	(1,18847.2)	0.0000	*
Concern COVID-19 (P)	Family	0.174	195.75	(1,18.1)	0.0000	*
Concern Child Safety (P)	Family	0.056	42.06	(1,18284.4)	0.0000	*
Concern Child Health (P)	Family	0.045	32.94	(1,15642.8)	0.0000	*
Concern Child Approach (P)	Family	0.035	5.94	(1,13.6)	0.0292	
Concern Own Possible Illness (P)	Family	0.066	14.64	(1,16.7)	0.0014	*
Anxiety (C)	Child	0.114	179.87	(1,3632.3)	0.0000	*
Time:Diagnosis (C)	Base	0.080	0.62	(12,11314.7)	0.8302	
Time:Days Since Pandemic	Base	-0.069	14.80	(2,32.4)	0.0000	*
Time:Anxiety Disorder (P)	Base	-0.103	64.70	(2,13480.8)	0.0000	*

Time:Relation to Child	Base	-0.058	2.50	(4,7284.3)	0.0404	
Time:Days Since National Peak	Base	0.019	3.19	(2,38.8)	0.0521	
Time:Age(C)	Base	0.017	1.44	(2,11071.9)	0.2363	
Time:Concern Illness (P)	Family	0.133	40.82	(2,17188.3)	0.0000	*
Time:Concern COVID-19 (P)	Family	0.194	96.69	(2,16915.2)	0.0000	*
Time:Anxiety(C)	Child	-0.060	18.29	(2,15208.1)	0.0000	*

Table S3B. Type II ANOVA breakdown of the final multilevel regression for Child Anxiety. Max. Beta indicates the maximum standardized contrast among pairwise differences. * = $p < 0.005$

**Effect**	**Block**	**Max. Beta**	***F***	**DF**	***p***	
Age (P)	Base	-0.011	1.42	(1,5468.8)	0.2339	
Sex (P)	Base	0.042	11.60	(1,4778.7)	0.0007	*
Relation to child	Base	0.122	3.46	(2,5019.4)	0.0316	
Education	Base	0.200	3.15	(5,4575)	0.0076	
Work Situation (P)	Base	0.263	1.80	(7,4502.9)	0.0827	
Anxiety Disorder (P)	Base	-0.098	13.26	(1,4930.5)	0.0003	*
Days Since Pandemic	Base	0.119	8.76	(1,199.3)	0.0035	*
Days Since National Peak	Base	-0.057	3.07	(1,127.1)	0.0824	
Diagnosis (C)	Base	-0.154	9.90	(6,13510.4)	0.0000	*
Age (C)	Base	0.008	0.76	(1,8773.4)	0.3833	
Sex (C)	Base	0.005	0.21	(1,15)	0.6539	
Anxiety Disorder (C)	Base	0.271	125.51	(1,22.7)	0.0000	*
Time	Base	0.087	12.20	(2,27.4)	0.0002	*
Cancellation of Public Events	Country	0.013	1.29	(1,66.6)	0.2599	
Obesity	Country	0.075	22.11	(1,69)	0.0000	*
Airports	Country	0.004	0.13	(1,55.3)	0.7176	
Anxiety (P)	Family	0.120	74.12	(1,17.6)	0.0000	*
Concern Ability	Family	0.038	20.92	(1,16335)	0.0000	*
Concern Possible Illness (P)	Family	-0.054	2.95	(1,17233.2)	0.0861	
Concern Family Conflict (P)	Family	-0.050	0.88	(1,12756.1)	0.3484	
Concern Motivation (P)	Family	0.043	11.32	(1,14.8)	0.0043	
Concern Illness (C)	Child	0.177	93.27	(1,25.5)	0.0000	*
Concern COVID-19 (C)	Child	0.196	720.62	(1,18287.5)	0.0000	*
Concern Safety (C)	Child	0.017	2.33	(1,16.7)	0.1458	
Concern Routine (C)	Child	0.135	241.93	(1,18764.6)	0.0000	*
Concern Family Conflict (C)	Child	0.052	66.87	(1,16883.2)	0.0000	*
Time:Diagnosis (C)	Base	-0.059	1.05	(12,12204.6)	0.3988	
Time:Days Since Pandemic	Base	0.017	1.57	(2,180.8)	0.2101	
Time:Age (C)	Base	0.012	1.34	(2,10070.4)	0.2617	
Time:Anxiety Disorder (P)	Base	-0.008	0.48	(2,13248.9)	0.6183	
Diagnosis (C):Days Since Pandemic	Base	-0.285	6.38	(6,11033.8)	0.0000	*

Diagnosis (C):Days Since National Peak	Base	0.187	7.23	(6,9613.2)	0.0000	*
Diagnosis (C):Education	Base	-0.532	2.85	(30,12993.7)	0.0000	*
Diagnosis (C):Anxiety Disorder (P)	Base	0.120	3.25	(6,14062.1)	0.0034	*
Diagnosis (C):Age (C)	Base	0.109	2.75	(6,12734.2)	0.0114	
Diagnosis (C):Work Situation (P)	Base	-0.531	1.78	(42,11928.9)	0.0015	*
Diagnosis (C):Sex (P)	Base	-0.141	2.10	(6,12988.7)	0.0501	
Diagnosis (C):Anxiety Disorder (C)	Base	0.066	4.61	(6,13533.2)	0.0001	*
Time:Anxiey (P)	Family	-0.031	6.86	(2,14793.1)	0.0010	*
Time:Concern Ability (P)	Family	0.023	2.46	(2,14470)	0.0859	
Time:Concern Possible Illness (P)	Family	0.016	0.97	(2,14681.8)	0.3776	
Diagnosis (C):Anxiety (P)	Family	-0.031	1.29	(6,12998.7)	0.2575	
Diagnosis (C):Concern Child Ability (P)	Family	0.034	5.78	(6,18641.1)	0.0000	*
Diagnosis (C):Concern Own Possible Illness (P)	Family	0.071	3.47	(6,18543.7)	0.0020	*
Diagnosis (C):Concern Family Conflict (P)	Family	0.093	1.94	(6,17551.1)	0.0701	
Time:Concern COVID-19 (C)	Child	0.112	60.93	(2,14423.5)	0.0000	*
Diagnosis (C):Concern Illness (C)	Child	0.058	2.62	(6,16520.4)	0.0153	
Diagnosis (C):Concern COVID-19 (C)	Child	-0.099	5.97	(6,18543.1)	0.0000	*
Diagnosis (C):Concern Safety (C)	Child	0.060	3.18	(6,15882.8)	0.0040	*
Diagnosis (C):Concern Routine (C)	Child	-0.072	4.57	(6,18628.2)	0.0001	*

Supplementary Materials B

B.1 Data analysis plan

Analysis of the data consisted of two general steps, **(1)** pre-processing, and **(2)** stepwise multilevel regressions of parent and child anxiety. These two general steps consisted of further specific steps, as summarized visually in Figure B1.

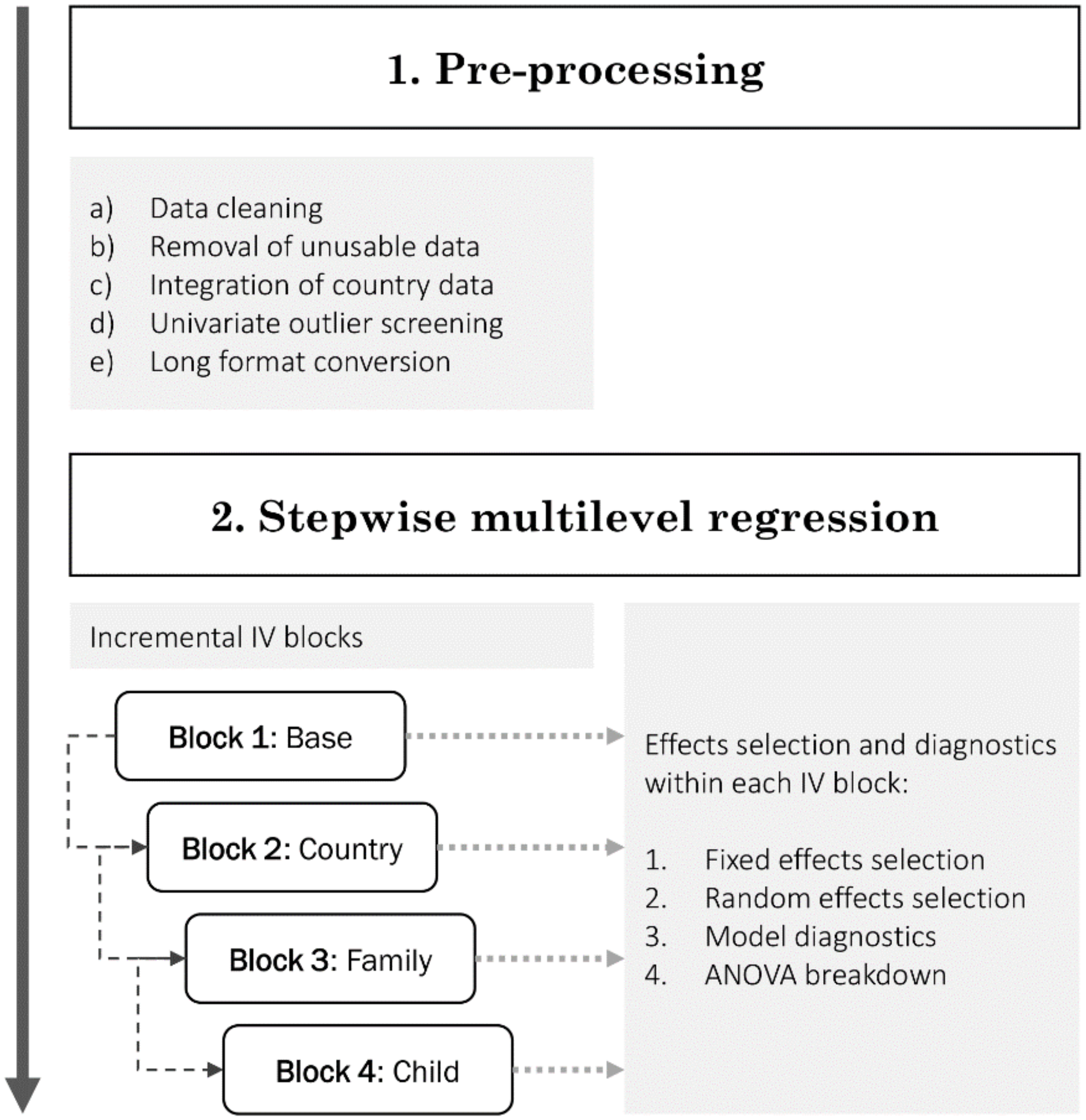

Figure B1. Pipeline of data analysis, consisting of (1) pre-processing followed by (2) stepwise multilevel regressions for parent and child anxiety. IV = independent variables.

B.2 Pre-processing

First, all country samples were merged and cleaned for language, notation, and formatting consistency. Second, respondents and variables that were unusable for analysis were removed a priori. This removal included (a) respondents that disagreed to have their data analyzed, (b) respondents that completed less than 25% of the questionnaire, (c) respondents that failed a check that separated human users from automated bots, (d) respondents for which the primary diagnosis of their NDCs child was missing, (e) variables that contained open text responses or raw dates, and (f) variables that had over 70% of their observations missing. This exclusion reduced the number of families from 22,741 to 8,043, the number of countries from 128 to 78, and the number of variables from 406 to 268.

Third, country variables were added to the cleaned data that measured national COVID19 evolution, national government responses to the pandemic, and structural

country descriptors (e.g., size, population). COVID19 evolution statistics during the time of the study (until 31.07.2020) were sourced from the European Center for Disease Prevention and Control.[1] From these statistics, we calculated for each country total deaths, total deaths per million inhabitants, peak deaths, peak deaths per million, date of the first death, date of peak deaths per million. Peak deaths and peak deaths per million were calculated from a 7-day moving average of deaths per million, to reduce the impact of outlying values. From the calculated evolution statistics, two new variables were derived to express for each family their distance to the international pandemic start, and their distance to their national pandemic peak. Distance to the international pandemic start was calculated as the difference in days between 11.03.2020[2] and the day the family completed the questionnaire (i.e., the "now" moment). Distance to the national pandemic peak was calculated as the difference in days between the day peak deaths were achieved nationally and the day the family completed the questionnaire.[3] Multilevel regressions subsequently controlled for these two distance variables, to adjust for the fact that different families completed the questionnaire at different times during the pandemic.

Government response statistics were sourced from the Oxford COVID-19 Government Response Tracker (Hale et al., 2021[4]), which included the tracked government response to the pandemic in multiple areas (e.g., public transport, teleworking, financial aid) and four aggregated indexes as general measures of response severity (including the stringency index - containment and closure policies, sometimes referred to as lockdown policies). Structural country descriptors were sourced from the CIA World Factbook,[5] and included variables on geographic, demographic, economic and political aspects of each country.

Fourth, a univariate outlier screening was conducted on the remaining data to check for impossible or unusual values. For categorical variables, levels with low occurrence were identified as outlying. For continuous variables, boxplots were used to identify observations beyond 1.5 times the interquartile distance of the rank-ordered data. Impossible values were converted to missing, while outlying values were simply flagged for later analyses.

Fifth, the pre-processed and expanded data were restructured from "wide format" to "long format" for compatibility with multilevel regression. In long format, each respondent/family had between 3 or 6 rows of repeated measures data, corresponding to the child (NDCs and TD) × time (before, start, now) repeated measures structure. As such, the full hierarchical structure of the data consisted of time points (before, start, now) nested in children (NDCs and TD), children nested in families, and families nested in countries. The presence of this data hierarchy motivated the use of multilevel models for stepwise analysis.

B.3 Stepwise multilevel regression

The final long-format data were entered into two stepwise multilevel regressions, with as dependent variables (DV) parent anxiety and child anxiety, respectively. For both DVs, modelling proceeded identically as a blocked stepwise regression search. Four blocks of independent variables (IVs) were added incrementally to obtain a final model, (1) base variables, (2) country variables, (3) family variables, and (4) child variables. The base model included the primary design IVs, which were the diagnosis group variable (ASD = Autism Spectrum Disorder, ADHD = Attention-Deficit/Hyperactivity Disorder, DLD = Developmental Language Disorder, DS = Down syndrome, WS = Williams syndrome, ID = Intellectual Disability-Not otherwise specified, TDS = Typically developing sibling) interacting with categorical time ("before", "start", "now"). As well, the base model

included controlling covariates for demographical parent and child aspects (e.g., age, gender), each family's distance to the international pandemic start, and the distance to their national pandemic peak. Subsequent IV blocks add further predictors of anxiety going from the macro-level of influence (i.e., country aspects) to the micro-level of influence (i.e., child aspects). The full list of variables for blocks 1–4 can be found in Table 2 in the main paper.

For each block, fitting the multilevel model consisted of four steps, (a) fixed effects selection, (b) random effects selection, (c) model diagnostics and inspection, and (d) ANOVA breakdown of effects. However, step (a) was omitted for the base model, due to these IVs representing structural information that we wished to control for, no matter its statistical significance.

Fixed effects selection. Within blocks 2–4, important fixed effects were identified by a forward selection pass, followed by backward elimination pass:

- *Forward selection pass*: At each step of the pass, the IV entered the model with the lowest *p*-value that surpassed 0.005 in significance. This was repeated until no new IV reached the threshold for inclusion.
- *Backward elimination pass*: At each step of the pass, the IV in the model with the highest p-value that was less significant than 0.05 was removed. This was repeated until no further IV in the model reached the threshold for elimination.

For the final set of selected IVs within each block, we further investigated possible two-way interactions between the selected IVs and either time or diagnosis group.

Random effects selection. Once the important fixed effects had been identified in a block, important random effects were identified in that block. Random effects in a multilevel model reflect different sources of sampling error (e.g., repeated sampling of populations such as countries and families) and enabled us to consider the hierarchical nesting in the data. For block 1, we considered a base random effects structure consisting of a random intercept for countries and a random intercept for families, as well as random slopes for categorical time within those two intercepts. This base structure accounted for the possibility that there were baseline group and time differences due to random sampling of families and countries and was not subjected to further reduction. For blocks 2–4, important random slopes were identified with a stepwise forward and backward search, identical to the strategy conducted for the fixed effects. This time, Akaike's Information Criterion (AIC) was used as the criterion to add or remove random effects, with a relative increase or decrease in more than 2 AIC points considered as the threshold. AIC selection was preferred for the random effects due to complications related to inferential tests on variance parameters (see Fitzmaurice, Laird & Ware, 2004).

Model diagnostics. The model that was retained for each block by the fixed and random effects selection was first checked for its stability and statistical assumptions by inspecting (a) collinearity diagnostics, (b) influence diagnostics, and (c) residual diagnostics (linearity, normality, and homoscedasticity). For collinearity, we inspected variance inflation factors (VIF) for fixed effects, removing any IVs with a VIF that exceeded 10 (Kutner et al., 2005). For influence diagnostics, we inspected boxplots of model residuals and random effects, the former for identifying outlying cases, the latter for identifying outlying levels of the data hierarchy (e.g., outlying countries). For residual diagnostics, we first computed decorrelated residuals according to the procedure recommended by Fitzmaurice, Laird, and Ware (2004). Then, we plotted

these transformed residuals against transformed fitted values to check for issues with non-normality, non-constant variance, and non-linear associations.

ANOVA breakdown. Once a model had been diagnosed to be stable in a block, a Type II ANOVA breakdown using *F*-tests was calculated to inspect statistically significant effects. For multi-parameter *F*-tests (e.g., diagnosis group effect), pairwise contrasts were calculated using *t*-tests. All inferential tests were conducted at a reduced significance level of α = 0.001, following recent recommendations to reduce the rate of false positives in social sciences research (Benjamin et al., 2018). Degrees of freedom for inferential *F*- and *t*-tests were corrected for the presence of random effects according to the method of Satterthwaite (Fitzmaurice et al., 2004), which yields appropriate fractional degrees of freedom.

As a measure for goodness-of-fit, we calculated marginal R^2 for each added block of IVs. This allowed us to quantify cumulative proportion of (fixed) variance explained by each incrementally added block of IVs (i.e., country, family, child). As a measure of effect size, we calculated standardized regression coefficients for all effects.[6] For effects with more than two categorical levels, we selected the coefficient that captured the largest standardized difference observed between two levels (Maxwell & Delaney, 2004). Finally, effect plots were generated to visualize relevant interactions and main effects.

B.4 Software

All pre-processing and analyses were conducted using the R statistical software version 4.0.3 (R Core Team, 2020), with packages “mice” for analysis of missing data patterns (van Buuren & Groothuis-Oudshoorn, 2011), “lme4” and “lmerTest” for multilevel regression (Bates, Maechler, Bolker & Walker, 2015; Kuznetsova, Brockhoff & Christensens, 2017), “car” for model diagnostics (Fox & Weisberg, 2018), “r2glmm” for calculation of marginal R^2 goodness-of-fit (Jaeger, 2017), “effectsize” for standardized regression parameters (Ben-Shachar, Makowski, & Lüdecke, 2020), and “visreg” for visualization of model effects (Breheny & Burchett, 2017).

References

Bates, D., Maechler, M., Bolker, B., & Walker, S. (2015). Fitting Linear Mixed-Effects Models Using lme4. *Journal of Statistical Software*, *67(1)*, 1–48.

Ben-Shachar, M.S., Makowski, D., & Lüdecke, D. (2020). Compute and interpret indices of effect size. R package version 0.4.0.

Benjamin, D. J., Berger, J. O., Johannesson, M., Nosek, B. A., Wagenmakers, E. J., Berk, R., ... & Johnson, V. E. (2018). Redefine statistical significance. *Nature Human Behaviour*, *2(1)*, 6–10.

Breheny, P., & Burchett, W. (2017). Visualization of Regression Models Using visreg. *The R Journal*, *9*, 56–71.

Fitzmaurice, G.M., Laird, N.M., & Ware, J.H. (2012). *Applied Longitudinal Analysis*. John Wiley & Sons.

Fox, J., & Weisberg, S. (2019). *An R Companion to Applied Regression* (Third Edition). Thousand Oaks CA: Sage.

Hale, T., Angrist, N., Goldszmidt, R., Kira, B., Petherick, A., Phillips, T., Webster, S., Cameron-Blake, E., Hallas, L., Majumdar, S., & Tatlow, H. (2021). A global panel database of pandemic policies (Oxford COVID-19 Government Response Tracker). *Nature Human Behaviour*, *5*, 529–538.

Jaeger, B. (2017). r2glmm: Computes R Squared for Mixed (Multilevel) Models. R package version 0.1.2.

Kutner, M.H., Nachtsheim, C.J., Neter, J., & Li, W. (2005). *Applied Linear Statistical Models*. Boston: McGraw-Hill Irwin.

Kuznetsova A, Brockhoff, P.B., & Christensen, R.H.B. (2017). “lmerTest Package: Tests in Linear Mixed Effects Models.” *Journal of Statistical Software*, *82(13)*, 1–26.

Maxwell, S. E., & Delaney, H. D. (2004). *Designing Experiments and Analyzing* Data: A model comparison perspective. Erlbaum. Mahwah, NJ.

R Core Team (2020). R: A language and environment for statistical computing. R Foundation for Statistical Computing, Vienna, Austria.

Rights, J. D., & Sterba, S. K. (2019). Quantifying explained variance in multilevel models: An integrative framework for defining R-squared measures. Psychological Methods, 24(3), 309–338. https://doi.org/10.1037/met0000184

van Buuren, S., & Groothuis-Oudshoorn, K. (2011). mice: Multivariate imputation by chained equations in R. Journal of Statistical Software, 45(3), 1–67. https://doi.org/10.18637/jss.v045.i03
